# The Anaesthesiologist and Palliative Care in a Newborn with the Adam “Sequence”

**DOI:** 10.1155/2017/6230923

**Published:** 2017-02-23

**Authors:** Alberto Vieira Pantoja, Maria Emília Gonçalves Estevez, Bruno Lima Pessoa, Fernando de Paiva Araújo, Bruno Mendonça Barcellos, Ciro Augusto Floriani, Marco Antonio Cardoso de Resende

**Affiliations:** ^1^Fluminense Federal University (UFF), Niteroi, RJ, Brazil; ^2^National Institute of Traumatology and Orthopaedics (INTO), Rio de Janeiro, RJ, Brazil; ^3^Monte Sinai Hospital, Juiz de Fora, MG, Brazil; ^4^National School of Public Health of the Osvaldo Cruz Foundation (ENSP-FOC), Rio de Janeiro, RJ, Brazil

## Abstract

Reports focusing on biomedical principlism and the role of anaesthesiologists in palliative care are rare. We present the case of a newborn with multiple craniofacial anomalies and a diagnosis of ADAM “sequence,” in which surgical removal of placental adhesions to the dura mater and the correction of meningocele was not indicated due to the very short life expectancy. After 48 hours, the odor from the placenta indicted a necrotic process, which prevented the parents from being close to the child and increased his isolation. Urgent surgery was performed, after which the newborn was transported to the ICU and intubated under controlled mechanical ventilation. The patient died a week later. The principles of beneficence, nonmaleficence, justice, and respect for autonomy are simultaneously an inspiratory and regulatory framework for clinical practice. Although only necessary procedures are defended, which suggests a position contrary to invasive interventions at the end of life, sometimes they are the best palliative measures that can be taken in cases like the one described here.

## 1. Introduction

The maintenance of life is historically the foundation of medical activity. However, scientific and technological developments lead to potential extremes that elicit questions concerning dignity at the end of life. Ethical decision-making cannot be conceived without propaedeutic and therapeutic details and the active and autonomous participation of the patient and/or their family. In this context, the doctor is inserted into a philosophical debate, which outperforms the technical possibilities, far from the protocols. Although dedicated to ethical values and practices, he is sometimes required to set limits on life support. Following the indication of surgical treatment, the anaesthesiologist's activity assumes the condition of intermediary, involving the patient and the surgeon, who eventually relates the patient to the hospital structure itself.

Reports that focus on biomedical principlism and the role of anaesthesiologists in palliative care in neonates are rare. We found it difficult to precisely define the boundary between what should be done and what could seem unnecessary. We report the case of a neonate with ADAM “sequence,” showing frontal, temporal, and parietal cranial agenesis, facial cleft, encephalomeningocele, and placental adhesions to the dura mater, by which surgical removal was initially contraindicated due to the minimum duration of patient's survival.

## 2. Case Presentation

A two-day-old male newborn, weighing 3900 g, born by Caesarean section, Apgar score 6–9, Capurro assessment of 41 weeks and 6 days, presented with multiple craniofacial anomalies (hypertelorism, agenesis of eyelids, facial cleft, cleft lip, and palate), bilateral frontal parietal encephalocele, failed cerebral hemisphere separation and placental adhesions to the dura mater, and pseudosyndactyly on the right hand. The ultrasound exam at 20 weeks had already indicated possible brain malformation. At birth, he was diagnosed with a probable ADAM “sequence” and his prognosis was a life expectancy of fewer than 24 hours. Thus, removal of the placental adhesions from the dura mater and the correction of meningocele were not indicated. However, after 48 hours, the odour from the placenta indicated a necrotic process, preventing the parents from being close to the child and increasing his isolation, so an urgent surgery was requested. The newborn was eupneic without supplemental oxygen when he arrived at the operating room and hemodynamically stable without vasoactive amines. He was monitored with a precordial stethoscope, ECG in DII, noninvasive blood pressure, and rectal thermometer and actively heated by a thermal mattress. Preoxygenation was performed under a facial mask, and then pure inhalational induction with sevoflurane 5 vol% under spontaneous ventilation was used to achieve adequate anaesthesia. Venipuncture with a 22 G cannula in the left forearm and tracheal intubation tube with 3.5 MMDI were performed. Anaesthesia was maintained with 0.4 FiO_2_ and 2-3 vol% sevoflurane and titrated fentanyl up to a total dose of 15 *µ*g, under controlled ventilation, in PCV (15 cm H_2_O) with PEEP (5 cm H_2_O) in a CO_2_ absorber system. He was hydrated with 130 mL of 0.9% NaCl. During the procedure, no hemodynamic instability or other significant complications were observed. After surgery had ended (95 min), the intubated newborn was transferred to the ICU, under controlled ventilation, and died a week later. Importantly, his parents signed a term of free, informed consent.

## 3. Discussion

ADAM sequence is an uncommon congenital disorder with highly variable spectrum. It comprises a series of craniofacial, thoracic, abdominal, and limb malformations in different proportions in association with amniotic bands [1, 2]. In the literature, this condition is also known as ADAM complex, amniotic band sequence, Streeter's dysplasia, congenital constriction bands, and pseudoainhum [3]. ADAM is an acronym for amniotic deformities, adhesions, and mutilations. It remains as a causal and pathogenetic enigma. Among the major diagnostic criteria are amniotic adhesions anywhere on body usually with associated disruptions of limb(s) and/or trunk and /or head; pseudosyndactyly; ring constrictions of limbs; apparent amputations; variable internal primary malformations [4].

The ADAM sequence occurs in 1 in 1,200–15,000 in live births [1, 5, 6] and 1 : 70 in stillbirths [7]. It affects both genders in the same proportion. Clinically it may present as a minor digital abnormality or limb malformation, with syndactyly or clubfoot, but also as autoamputation and catastrophic craniofacial deformities [8, 9]. Importantly, often times are incompatible with life, as in our case here reported.

**Figure 1:**
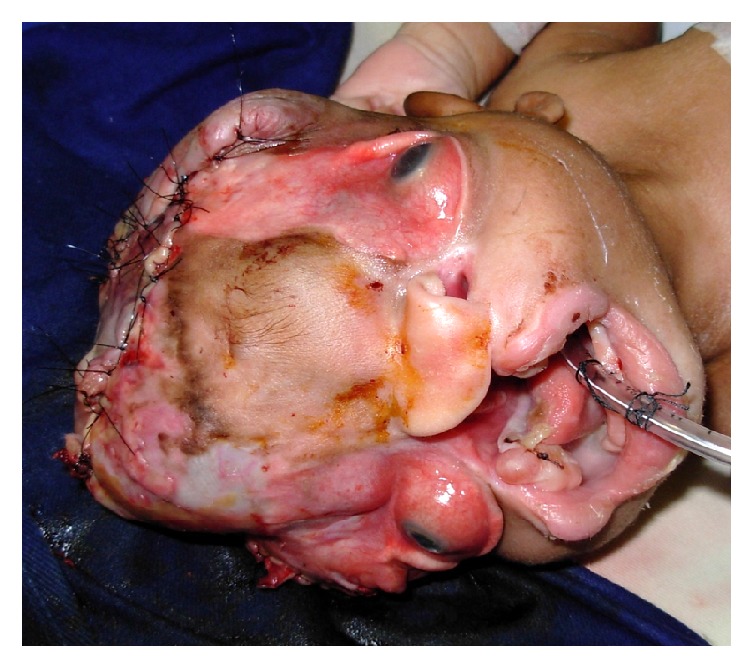
After surgical intervention.

Craniofacial anomalies and brain deformities are a challenge for the anaesthesiologist. The known risks include difficult airway management, undiagnosed malformations (particularly in the cardiovascular system), significant hypovolemia disproportional to intraoperative bleeding, and prolonged brain exposure. Hypothermia and secondary complications when handling vital brain structures may be exacerbated. In this case, the facial anomaly (Figure 1) prevented from mask ventilation, so inhalational induction was the chosen approach to preserving spontaneous ventilation. The main demands the team faced were the desires of the family, the commitment to life, and the dignity of death in children with poor prognosis. Understanding the relationship between patients and physicians as the core of medical ethics, Truog sets out the evolution of the same in three interrelated spheres—clinical care, research, and society [10]. The behaviour of anaesthesiologists, indeed any medical professional, should value the biomedical principialist approach, despite its potential deficiencies, in which the principles of beneficence, nonmaleficence, justice, and respect for autonomy are simultaneously an inspiratory and regulatory framework for clinical practice [11].

Nonmaleficence, the oldest of the four principles of biomedical ethics—*primum non nocere*—, was observed through the desire of not leaving the placental material attached to the newborn during the process of necrosis. This fact mobilized the parents and pediatrician to request urgent surgery and facilitated some decisions. Beneficence was acted on when preparing the newborn with the most appropriate anaesthesia and proceeding to clean the necrotic area. The surgery was also useful measure for others since it enabled the parents to be close to the newborn and resolved the isolation caused by the strong odor. The principle of justice can also be observed in this case, since the hospital structure was placed at the service of a profoundly vulnerable human being in their nascence and, paradoxically, at the end of life. This ensured equitable access to resources, that is, providing the best conditions available to those with limited access. Although the principle of autonomy cannot be directly applied to the newborn, the parents' wishes were addressed. In other words, all the decisions that the team had made were what the parents expected to be done.

In Brazil, unilateral decisions taken by the physicians, regarding not to interfere, can be registered in the medical records without the permission or notification of the family. However, it should be clear that such measures are not intended to prolong patient suffering before the inevitable outcome. In paediatrics, in particular, this approach implies the risk of not having the parents' approval, and that such decisions may result in errors in the survival prognosis [12]. It should be addressed that the team was attentive to the plight of the newborn and his parents, with no error occurring concerning his prognosis. Yet the mobilizing force was driven by the understanding that caring for this newborn should have been done according to the reconsideration about the initial decision of not to operate him on. In the end, all the conditions needed for the team to care for him until his death were reached.

One important aspect that permitted the care provided in this case is that there was no contradiction between the practice of care and the use of high technology. Our understanding is that, even when defending necessary procedures only, which suggests a position contrary to invasive interventions at the end of life, sometimes they are the best palliative measures that might be taken in cases like the one described here. Importantly, the understanding between the family and the medical team was established, with the outcome showing that the approach of surgical intervention—initially discarded—was the most appropriate. Thus, we argue that before and after birth and when the outcome is uncertain, all available options and detailed information should be fully explained to the family members.

The role of anaesthesiologists goes beyond of relieving pain, and it is important to invite them to participate in decisions like the case in question, actively. The foundations of such cases must be established based on the best available reasoning and not on individual emotions and valuations [13]. Altogether, our approach for the case represented all the principles mentioned earlier.
